# Dietary Cinnamon Successfully Enhanced the Growth Performance, Growth Hormone, Antibacterial Capacity, and Immunity of European Sea Bass (*Dicentrarchus labrax*)

**DOI:** 10.3390/ani11072128

**Published:** 2021-07-17

**Authors:** Mahmoud M. Habiba, Ebtehal E. Hussein, Ahmed M. Ashry, Ahmed M. El-Zayat, Aziza M. Hassan, Ahmed M. El-Shehawi, Hani Sewilam, Hien Van Doan, Mahmoud A.O. Dawood

**Affiliations:** 1 National Institute of Oceanography and Fisheries, NIOF, Cairo 11865, Egypt; mahmoudhabiba40@yahoo.com (M.M.H.); ahmed_ashry74@yahoo.com (A.M.A.); 2 Department of Poultry and Fish Production, Faculty of Agriculture, Menoufia University, Shebin El-Kom 32516, Egypt; ebtehal.elsaid@yahoo.com; 3 Department of Fish Production, Faculty of Agriculture, Al-Azhar University, Nasr City, Cairo 11865, Egypt; Ahmedelzayat.5@azhar.edu.eg; 4 Department of Biotechnology, College of Science, Taif University, Taif P.O. Box 11099, Saudi Arabia; a.hasn@tu.edu.sa (A.M.H.); elshehawi@hotmail.com (A.M.E.-S.); 5 The Center for Applied Research on the Environment and Sustainability, The American University in Cairo, Cairo 11835, Egypt; sewilam@aucegypt.edu; 6 Department of Engineering Hydrology, RWTH Aachen University, 52062 Aachen, Germany; 7 Department of Animal and Aquatic Sciences, Faculty of Agriculture, Chiang Mai University, Chiang Mai 50200, Thailand; 8 Innovative Agriculture Research Center, Faculty of Agriculture, Chiang Mai University, Chiang Mai 50200, Thailand; 9 Department of Animal Production, Faculty of Agriculture, Kafrelsheikh University, Kafrelsheikh 33512, Egypt

**Keywords:** sustainable aquaculture, feed additives, wellbeing, health condition, spices

## Abstract

**Simple Summary:**

Optimum aquafeed formulations should consider incorporating both nutritional and non-nutritional substances to fulfill the basal requirements and achieve the welfare of aquatic animals. In this context, medicinal plants are validated for their functionality as non-chemical derived materials. This study evaluated dietary cinnamon at varying levels on the growth performance and health status of European sea bass. The results showed the positive influence of the inclusion of cinnamon powder in the diets for European sea bass on the growth performance, feed utilization, blood analysis, and intestinal microbial community. Therefore, it can be concluded that 10–15 g/kg of cinnamon powder is suggested with no adverse effects for better performance of European sea bass.

**Abstract:**

Dietary cinnamon has several bioactive compounds with growth-promoting and immunomodulation potential and is suggested for finfish species. This study evaluated the inclusion of cinnamon at 0, 10, 15, and 20 g/kg in European sea bass (*Dicentrarchus labrax*) diets. After 90 days, the highest final weight, weight gain, specific growth rate, protein efficiency ratio, and the lowest feed conversion ratio were seen in fish treated with 10 g/kg (*p* < 0.05). Further, the measured growth hormone in the blood indicated that fish treated with 10 g/kg had a higher level than fish 0 and 20 g/kg. After the feeding trial, fish treated with cinnamon at varying levels had higher lipid content than fish before the feeding trial (*p* < 0.05). Lower *Vibrio* spp. and Faecal Coliform counts were observed in fish treated with cinnamon than fish fed a cinnamon-free diet (*p* < 0.05). The hematocrit level was markedly (*p* < 0.05) increased in fish fed cinnamon at 10 g/kg compared to the control without significant differences with fish fed 15 and 20 g/kg. Hemoglobin was significantly increased in fish treated with cinnamon at 10, 15, and 20 g/kg compared to fish fed a cinnamon-free diet (*p* < 0.05). Red and white blood cells (RBCs and WBCs) were meaningfully (*p* < 0.05) increased in fish treated with cinnamon compared with the control. Markedly, fish treated with cinnamon had higher serum total lipids than the control with the highest value in fish treated with 15 g/kg (*p* < 0.05). The lysozyme activity was markedly higher in fish treated with 15 g cinnamon/kg than fish fed 0, 10, and 20 g/kg (*p* < 0.05). Moreover, phagocytic activity was significantly higher in fish treated with cinnamon at 10, and 15 g/kg than fish fed 0 and 20 g/kg (*p* < 0.05). In conclusion, dietary cinnamon is suggested at 10–15 g/kg for achieving the high production and wellbeing of European sea bass.

## 1. Introduction

Aquaculture activity is one of the leading food sources and incomes in the world [1,2]. It is an aim to grow aquatic animals under well-controlled conditions to have the optimal growth performance and productivity within the shortest possible time [3,4]. In this way, aquaculture provides humanity with enough seafood that should not be compromised with stressful factors involved in increasing productivity [5,6]. The suitable farming conditions should consider the environmental factors (water quality, temperature, experience, and management), source of seeds, and quality of feeds, and the use of environmentally friendly additives known for their positive impact on aquatic animals’ wellbeing and health status [7,8,9]. Different supplements have been added to fish feed to enhance growth, health, immunity, and fish productivity [10,11]. It is common to add immunostimulatory additives as a constraint for organic aquaculture in the feed industry [12]. Natural supplements attracted the attention of academia and farmers because of their safety and effectiveness compared with synthetic products [13]. Further, medicinal plants are widely used in aquaculture for their growth-promoting, immunomodulation, and antibacterial effects [14,15]. Several medicinal plants were applied in aquacultures, such as garlic, onion, basil, cumin, anise, fenugreek, thyme, and paprika [16,17]. Recently, many investigations evaluated the possibility of using medicinal plants and extracts in aquafeed based on a species-specific manner [18,19,20,21].

Cinnamon (*Cinnamomum* sp.) is a well-known spicy plant that grows in tropical areas with a powerful potential as a growth promotor and immunostimulant agent [22]. Cinnamon contains several functional components, vitamins, minerals, phenolic compounds (tannins, saponins), and essential oils (cinnamic aldehyde and cinnamyl aldehyde) [23,24,25]. Markedly, these components are involved in antibacterial, antineoplastic, cardiovascular, cholesterol-reducing, anti-inflammation, immunostimulant, and antioxidative effects [26]. The potential effects of cinnamon were investigated in humans, livestock, and poultry [22,27]. In aquaculture, the effects of cinnamon were evaluated in grass carp (*Ctenopharyngodon idella*) [28], rainbow trout (*Oncorhynchus mykiss*) [29], and Nile tilapia (*Oreochromis niloticus*) [27,30]. The results showed that cinnamon could improve growth performance, feed digestibility, immunity, antioxidative capacity, and resistance to pathogenic bacteria.

European sea bass (*Dicentrarchus labrax*) is a popular marine fish species and well accepted by consumers and stockholders [31]. The inclusion of medicinal plants in European sea bass feed is suggested to maintain high productivity and wellbeing. Considering the potential roles of cinnamon, this study aimed at testing the potential effect of cinnamon on the productivity of European sea bass. For this, the growth performance, blood health, immunity, and intestinal antibacterial capacity responses were tested.

## 2. Materials and Methods

### 2.1. Experimental Fish

Twenty fingerlings of European sea bass (*Dicentrarchus labrax*) were randomly selected with an average body weight of 7.08 ± 0.14 g and stocked in net enclosures (hapas) (12 hapas total; each 1 m × 1 m × 1 m). All hapas were fixed in four concrete tanks, each one of 1 m × 8 m × 3 m with three replicates each at El-Max station, the National Institute of Oceanography and Fisheries, Alexandria, Egypt. All procedures and handling of animals were conducted in compliance with the guidelines of the National Institute of Oceanography and Fisheries, Egypt. All tanks were washed cautiously, filled with salt water, and constantly aerated. The hapas were washed every week to remove residuals. At the same time, fish were kept in plastic buckets while hapas were washed with water then refixed inside the concrete tanks. Underground saltwater was used during the trial with a salinity of 32 ppt. Every three days, 10% of the water was replaced with clean seawater. All fish (total biomass per hapas) were weighted to check the growth and readjust the feed amount every two weeks. Water quality was checked during the trial and averaged (±SE): water temperature, 26.65 ± 0.2 °C; dissolved oxygen, 5.61 ± 0.05 mg/L; pH, 7.42 ± 0.02; ammonia, 0.02 ± 0.01 mg/L, and salinity, 32 ± 0.02 ppt. Triplicate groups were fed on the experimental diets up to satiation level three times per day (9:00 a.m., 11:00 a.m., and 1:00 p.m.) for 90 days.

### 2.2. Experimental Diets

The experimental diets were prepared to be similar in crude protein (46 ± 0.01%) and crude lipids (15.32 ± 0.04%) (Table 1). All the ingredients were purchased from a local store. Then, ingredients were first ground to small particle size (approximately 1 mm) in a Wiley mill (Labx Company, Midland, ON, Canada). Water was added to each diet and pelletized through the kitchen machine. The pellets were dried, preserved, and kept in sealed bags. The formulated diets were supplemented with powdered cinnamon bark (*Cinnamomum* sp.) at 0, 10, 15, and 20 g per kg diet. The cinnamon was obtained from the local market, and the inclusion levels were decided by following Ravardshiri, Bahram, Javadian and Bahrekazemi [29]. The composition and chemical analysis of formulated diets are shown in Table 1.

### 2.3. Body Composition Analysis

Twenty fish were collected before the feeding trial and immediately kept at −20 °C for initial body chemical composition. The moisture, protein, lipids, and ash contents of the fish body were also performed by the end of the feeding trial, according to AOAC [33]. Six fish from each treatment were randomly collected, then the whole fish was homogenized and kept at −20 °C for biochemical analysis at the research facility of the National Institute of Oceanography and Fisheries, Alexandria, Egypt. The dry matter, crude protein, and crude lipids were analyzed respectively after drying in a drying oven (105 °C for 24 h), by micro Kjeldahl (N × 6.25), and ether extraction by Soxhlet method. Fish growth parameters were calculated as gain (g/fish) (weight gain (WG) = final body weight (BW) − initial BW), specific growth rate (SGR = (ln final BW − ln initial BW) × 100/days), and survival = no. of fish at the end of the experiment/no. of fish at the start of the experiment × 100). In addition, feed utilization parameters such as feed conversion ratio (FCR; dry feed consumed/WG) and the protein efficiency ratio (PER; WG/protein intake) were determined. During the trial, fish were fed the test diets up to the satiation level, and the consumed feed was recorded to calculate the total feed intake at the end of the trial.

### 2.4. Hematological and Blood Biochemical Analysis

Another three fish from each hapa were collected (9 fish/group), and blood was obtained from the caudal vein using heparinized syringes (2.5 mL) for hematological analysis. In addition, non-heparinized syringes were used to collect blood for serum separation. The samples were left for 4 h at 4 °C, then centrifuged at 3000× *g* for 15 min at 4 °C. Then serum samples were collected and kept at −80 °C for further biochemical analysis. The white blood cell (WBC) and red blood cell (RBC) counts and hemoglobin concentration were undertaken following standard procedures [34]. Hematocrit was determined by the microhematocrit method, while the hemoglobin concentration was determined with a spectrophotometer (Model RA 1000, Technicon Corporation, Pittsburgh, Pennsylvania, USA) at 540 nm, using the Blaxhall and Daisley [35] method. The mean corpuscular volume (MCV), mean corpuscular hemoglobin (MCH), and mean corpuscular hemoglobin concentration (MCHC) were calculated by following Dacie and Lewis [36].

Serum alanine aminotransferase (ALT) and aspartate aminotransferase (AST) activities were detected by following the method of Reitman and Frankel [37]. Alkaline phosphatase (ALP) enzyme activity, urea, creatinine, and total lipids were determined using commercially supplied kits by Pasteur Lab (Diagnostics Pasteur, Marnes la Coquette, France) [38]. The growth hormone was estimated in the serum samples according to Lugo, et al. [39] using a non-competitive ELISA with 96-well MaxiSorp plates (Nalge Nunc International, Roskilde, Denmark). The absorbance was measured at 492 nm in a spectrophotometer (Titertek Multiskan Plus).

The leukocyte phagocytic function was determined following the method of Cai et al. [40]. The number of leukocytes that engulfed bacteria was counted as a percentage in relation to the total leukocyte number in the smear from the phagocytosis assay. By following Kawahara, et al. [41], the phagocytic activity and phagocytic index were determined. The lysozyme activity of serum was assayed according to the methods described by Demers and Bayne [42].

### 2.5. Intestinal Antimicrobial Analysis

The distal intestines were dissected from 3 fish per hapa and homogenized in 10 mL of 3% sterile sodium chloride solution. The homogenized samples were diluted from 10^−1^ to 10^−5^. Selective agar media were used to grow bacteria using 1 mL from diluted samples. The count of *Vibrio* spp. was determined by using thiosulfate-citrate-bile salt-sucrose (TCBS) agar [43]. For *Escherichia coli*, modified Fecal Coliform (mFC) agar was used (ISO (International Organization for Standardization) No. 9308/1, 1990). Incubation of the plates was carried out at 30 °C for 24–48 h for enumeration, except for the mFC medium, which was incubated at 44 °C for 24 h. Then de Man, Rogosa, and Sharpe (MRS) medium were used to cultivate the fermentative acid bacteria, which were incubated at 37 °C for 48 h under anaerobic conditions [44].

### 2.6. Statistical Analysis

Shapiro–Wilk and Levene tests confirmed a normal distribution and homogeneity of variance. Mean values and standard error (mean ± SE) for each parameter of all treatments were first calculated. The results were subjected to one analysis of variance (ANOVA) to test the effect of treatments on fish performance. Differences between means were compared using Duncan multiple range tests to test the significance level among means of treatments using SPSS (version 22) at *p* < 0.05. Significant differences were seen between the initial and the final body composition of fish fed varying levels of cinnamon, as shown by the *t*-test (*p* < 0.05).

## 3. Results

### 3.1. Growth Performance, Growth Hormone, and Carcass Composition

The final weight (FW), weight gain (WG), and protein efficiency rate (PER) were meaningfully (*p* < 0.05) enhanced by supplementing cinnamon at 10–15 g/kg (Table 2). Further, the specific growth rate (SGR) was markedly increased in fish treated with a 10 g cinnamon/kg diet compared to fish fed 0, 15, and 20 g/kg (*p* < 0.05). On the other hand, the feed conversion ratio was decreased in fish fed 10 and 15 g/kg. The highest FW, WG, SGR, PER, and the lowest FCR were seen in fish treated with 10 g/kg (*p* < 0.05) (Table 2).

**Figure 1:**
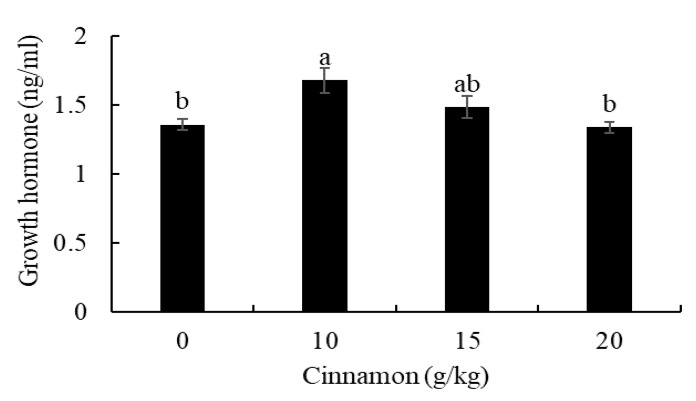
Blood growth hormone levels of European sea bass fed dietary cinnamon. Bars with different letters are significantly different (*p* < 0.05).

The measured growth hormone in the blood indicated that European sea bass treated with 10 g/kg had a higher level than fish 0 and 20 g/kg (Figure 1).

The body composition of fish at the end of the experiment was similar for all conditions (*p* > 0.05) but significantly higher than fish sampled at the beginning of the experiment (Table 3).

### 3.2. Intestine Antibacterial Capacity

**Figure 2:**
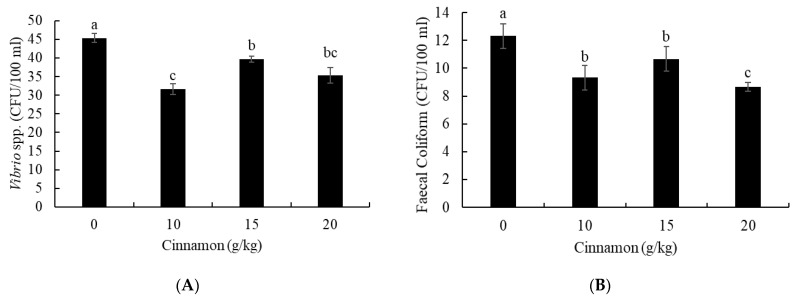
Intestinal harmful bacterial count: (**A**) *Vibrio* spp. and (**B**) Faecal Coliform of European seabass fed dietary cinnamon. Bars with different letters are significantly different (*p* < 0.05).

The effect of cinnamon on the antibacterial capacity of European sea bass was detected by *Vibrio* spp. and Faecal Coliform. The results showed lower *Vibrio* spp. (Figure 2A) and Faecal Coliform (Figure 2B) in fish treated with cinnamon than fish fed a cinnamon-free diet (*p* < 0.05). Fish treated with 10 g cinnamon/kg had lower *Vibrio* spp. count than fish treated with 15 g/kg without marked differences with fish treated with 20 g/kg (*p* < 0.05). Further, fish treated with 20 g cinnamon/kg had lower Faecal Coliform than fish treated with 10 and 15 g/kg (*p* < 0.05).

### 3.3. Hematology and Blood Biochemical Indices

The hematocrit level was markedly (*p* < 0.05) increased in fish fed cinnamon at 10 g/kg compared to the control without significant differences with fish fed 15 and 20 g/kg (Table 4). Hemoglobin was significantly increased in fish treated with cinnamon at 10, 15, and 20 g/kg compared to fish fed the cinnamon-free diet (*p* < 0.05) (Table 4). Red and white blood cells (RBCs and WBCs) were meaningfully (*p* < 0.05) increased in fish treated with cinnamon compared with the control (Table 4). Further, fish treated with 10 g/kg had higher WBCs than fish treated with 15 and 20 g/kg (*p* < 0.05). On the other hand, there were no significant differences between treatment for MCV, MCH, and MCHC. Furthermore, no marked effects were observed on the ALT, AST, ALP, urea, and creatinine in the serum samples of European sea bass treated with cinnamon at varying levels (*p* ˃ 0.05) (Table 5). Markedly, fish treated with cinnamon had higher serum total lipids than the control with the highest value in fish treated with 15 g/kg (*p* < 0.05) (Table 5).

### 3.4. Lysozyme and Phagocytic Activities

**Figure 3:**
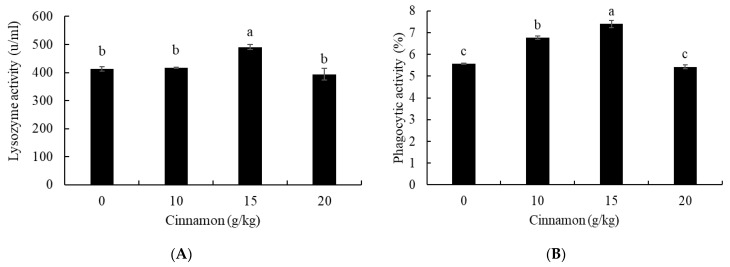
Blood lysozyme (**A**) and phagocytic (**B**) activities of European sea bass fed dietary cinnamon. Bars with different letters are significantly different (*p* < 0.05).

The lysozyme activity was markedly higher in European sea bass treated with 15 g cinnamon/kg than fish fed 0, 10, and 20 g/kg (*p* < 0.05) (Figure 3A). Moreover, phagocytic activity was significantly higher in fish treated with cinnamon at 10, and 15 g/kg than fish fed 0 and 20 g/kg (*p* < 0.05) (Figure 3B). Besides, fish fed 15 g cinnamon/kg had higher phagocytic activity than fish fed 10 g cinnamon/kg (*p* < 0.05).

## 4. Discussion

Cinnamon is another beneficial medicinal spice in the series of phytogenics involved in promoting growth performance, digestibility, antibacterial capacity, immunity, and wellbeing of aquatic animals [30]. Although cinnamon’s primary mode of action is not well documented in finfish species, cinnamon’s effect is well investigated in livestock, poultry, and human studies [22]. Cinnamon has several bioactive compounds such as vitamins, minerals, phenolic compounds (tannins, saponins), and essential oils (cinnamic aldehyde and cinnamyl aldehyde) [23,24,25] with high antibacterial capacity and antioxidative potential [45]. This results in the growth reduction of harmful bacteria, which negatively affect digestion and the local intestinal immunity. It was proved that trans-cinnamaldehyde showed antibacterial activity against pathogenic *Aeromonas* sp. [46]. Considering this hypothesis, the current study evaluated the intestinal antibacterial capacity that might be involved in enhancing European sea bass’s feed utilization and growth performance.

The results elucidated that European seabass treated with cinnamon had marked enhancement in the growth performance. The results are concurrent with several studies that investigated the growth-promoting role of cinnamon in finfish species. In Nile tilapia, Abdel-Tawwab, Samir, Abd El-Naby and Monier [30] reported that incorporating three grams of cinnamon/kg is recommended to enhance the growth performance. Further, rainbow trout fed dietary cinnamon at 30 g/kg led to enhanced growth performance [29]. The authors related the enhanced growth performance to activated digestive enzymes (protease, lipase, and amylase) in fish fed dietary cinnamon. The improvement in the digestion capacity is the main reason for increased growth performance [8]. Cinnamon has antibacterial components responsible for increasing the abundance of beneficial bacteria and reducing the pathogenic population leading to high secreted endogenous digestive enzymes [22]. In this context, the results illustrated that European seabass fed cinnamon had markedly improved feed conversion ratios and protein efficiency ratios, indicating high feed utilization. Similarly, Zhou, Jiang, Zhang, Feng, Wu, Liu, Jiang, Kuang, Tang and Peng [28] reported that grass carp treated with cinnamaldehyde had enhanced feed digestibility associated with high digestive enzyme activities. In poultry feeding, the inclusion of cinnamon resulted in similar enhanced feed utilization, which attributed to increased activity of digestive enzymes [47]. It is worth noting that the best growth performance and feed utilization in European seabass treated with cinnamon is seen in fish fed 10–15 g/kg. The inclusion of 20 g cinnamon/kg had no significant effects on the growth performance and feed utilization, confirming that cinnamon should be added based on a dose-dependent manner. It has been reported that including high levels of cinnamon could result in bitterness and astringency taste leading to low feed consumption [48].

The enhanced growth performance in this study can be attributed to increased growth hormone levels in the blood of European seabass treated with cinnamon. It has been hypothesized that cinnamon can increase the secretion of growth hormone from the pituitary gland by activating insulin-like growth factor (*IGF-1*) under the effect of cinnamaldehyde [49]. High expression of *IGF-1* led to the synthesis of proteins required to fulfill the primary metabolic function and build entire body tissues [50]. No previous studies have investigated the role of cinnamon in activating the growth hormone in fish, and further studies are recommended to reveal the relationship between increased growth hormone and cinnamon feeding in fish.

The decomposition of nutrients in fish bodies is usually affected by the feed composition, fish species, and rearing conditions [8]. The carcass composition of European sea bass treated with cinnamon was not markedly impacted after the feeding trial. However, lipid content was meaningfully increased in European sea bass after the feeding trial compared to before the feeding trial. After the feeding trial, the high lipid content in fish is probably related to increased final body weight compared to the initial body weight.

Medicinal plants are innovative antibacterial substances and help in regulating the diversity of intestinal microbiota [51,52]. The inclusion of cinnamon is an indicative model for confirming the antibacterial capacity in vivo and in vitro [53]. The results showed a meaningful reduction in the count of *Vibrio* spp. and Faecal Coliform in European seabass treated with cinnamon. Vibrio and Faecal Coliform bacteria are harmful to the intestinal local immunity and digestion capacity and reduce the digestion capacity and general health condition of fish [54]. This study showed enhanced growth performance and feed efficiency that might be explained by increased antibacterial capacity in European sea bass treated with cinnamon. The results agree with many studies that investigated the antibacterial role of cinnamon in vitro [51,55]. Cinnamon contains phenolic compounds, volatile components, and essential oils (e.g., cinnamaldehyde) manifested with antimicrobial and antifungal activities [56]. Cinnamon essential oil can disrupt the membranes of pathogenic bacteria [57]. Besides, cinnamon and its essential oils can improve intestinal mucus secretion, leading to reduced adhesion of harmful bacteria in the epithelial tissue [58]. Accordingly, the beneficial bacteria grow and show their effect in improving the digestion and absorption of feed in fish intestines [8].

Hematological and biochemical blood indices reflect the health status of fish when treated with various feed additives [59,60]. Although blood-related traits are quantitative and not involved in molecular analysis, they are easy to perform and understand by academia and farmers [61]. The results offered an enhanced hematological profile as indicated by the increased hemoglobin, hematocrit, RBCs, and WBCs in European sea bass treated with cinnamon. Usually, increased blood hemoglobin and RBCs indicate high Ferrin and low anemic features resulting in high respiration capacity [62]. Further, increased hematocrit and WBCs are associated with improved immunity and resistance to pathogenic infection [63]. The results agree with Ahmad, El Mesallamy, Samir and Zahran [27], who stated increased hemoglobin, hematocrit, RBCs, and WBCs in Nile tilapia fed a cinnamon diet. However, Ravardshiri, Bahram, Javadian and Bahrekazemi [29] reported no significant differences in the hemoglobin, hematocrit, RBCs, and WBCs values in rainbow trout treated with cinnamon. The discrepancies are probably attributed to the differences in fish species, feeding habits, duration of the trial, and level of cinnamon supplementation.

The absence of significant differences in total lipids, ALT, AST, ALP, urea, and creatinine levels refer to the stability of health status and absence of hepatic and renal failure in European sea bass treated with cinnamon. The results disagree with Ahmad, El Mesallamy, Samir and Zahran [27], who illustrated the beneficial effects of cinnamon on the blood biochemical profile of Nile tilapia, especially ALT, AST, and ALP. The discrepancies between the two studies are probably related to fish species, fish size, and experimental conditions. These results also confirm that cinnamon is recommended based on a species-specific manner. The results also showed that increased total blood lipids were seen in fish treated with cinnamon. The increased blood lipids are probably attributed to high feed utilization, thereby high lipid accumulation in the blood. Further future studies are required to clarify the role of cinnamon in increasing the total lipids in the blood of fish.

The performances of finfish species treated with medicinal plants can be evaluated by detecting the immune responses to predict how far fish can tolerate common infections, biotic and abiotic stressors [64,65]. In this context, the study showed improved immunity in European sea bass fed cinnamon as indicated by the activities of lysozyme and phagocytosis. Similarly, Abdel-Tawwab, Samir, Abd El-Naby and Monier [30] reported increased lysozyme activity in Nile tilapia fed cinnamon. The increased lysozyme activity refers to the ability of fish to hydrolysis the cell walls of harmful bacteria, while phagocytosis is a cellular immune defense against infection [66]. Cinnamon has an abundant amount of essential oils and phenolic substances involved in increasing the antibacterial capacity of fish [47]. Furthermore, enhanced immunity in fish is probably related to cinnamon’s antioxidative capacity, which reduces the impact of lipid peroxidation on the immune cells [29].

## 5. Conclusions

The results showed the positive influence of the inclusion of cinnamon powder in the diets of European sea bass on the growth performance, feed utilization, blood analysis, and intestinal microbial community. Therefore, it can be concluded that 10–15 g/kg of cinnamon powder is required with no adverse effects for better performances of European sea bass. No adverse effects were detected under the conditions tested, but the study was not extensive and long enough to determine if cinnamon negatively affects fish health in the long run. Further studies will be necessary to examine the cinnamon powder effect on the antioxidative capacity of European sea bass.

## Figures and Tables

**Table 1:** Formulation and chemical composition of the basal diet.

Ingredients (g/kg)	Cinnamon (g/kg)			
	0	10	15	20
Fish meal (67%)	400	400	400	400
Shrimp meal	110	110	110	110
Soybean (48%)	150	150	150	150
Yellow corn (7.5%)	50	50	50	50
Corn gluten (60%)	50	50	50	50
Wheat flour	100	90	85	80
Fish oil	100	100	100	100
Di-calcium phosphate	10	10	10	10
Vitamin and mineral premix ^1^	30	30	30	30
Cinnamon	0	10	15	20
Total	1000	1000	1000	1000
Chemical analysis				
Dry matter (%)	91.9	91.12	91.42	91.35
Crude protein (%)	46	45.99	46	45.99
Crude lipids (%)	15.28	15.3	15.33	15.37
Ash (%)	10.43	10.39	10.54	10.6
Crude fiber (%)	2.19	2.16	2.19	2.17
Nitrogen free extract	26.09	26.15	25.94	25.87
Gross energy (Kcal/100 g) ^2^	511.83	512.21	512.76	511.67

^1^ Vitamin and mineral mixture as previously detailed by Ashry, et al. [32]. ^2^ Gross energy based on protein (5.65 kcal/g), crude lipids (9.45 kcal/g), and nitrogen-free extract (4.12 kcal/g).

**Table 2:** Growth performance of European sea bass fed dietary cinnamon.

Item	Cinnamon (g/kg)			
	0	10	15	20
Initial weight (g)	7.22 ± 0.18	6.96 ± 0.09	7.05 ± 0.12	7.09 ± 0.38
Final weight (g)	38.84 ± 0.65 c	51.29 ± 1.45 a	42.25 ± 0.56 b	38.01 ± 1.14 c
Weight gain (g)	31.62 ± 0.72 c	44.33 ± 1.47 a	35.20 ± 0.64 b	30.92 ± 1.05 c
Specific growth rate (%/day)	1.87 ± 0.04 b	2.22 ± 0.04 a	1.99 ± 0.03 b	1.87 ± 0.06 b
Feed intake (g/fish)	63.92 ± 1.48	60.63 ± 1.20	58.15 ± 1.79	62.45 ± 1.66
Feed conversion ratio	2.02 ± 0.00 a	1.37 ± 0.02 c	1.65 ± 0.05 b	2.02 ± 0.03 a
Protein efficiency ratio	1.08 ± 0.00 c	1.59 ± 0.03 a	1.32 ± 0.04 b	1.08 ± 0.01 c
Survival (%)	91.67 ± 4.41	91.67 ± 4.41	93.33 ± 1.67	91.67 ± 4.41

Values in the same row with different letters are significantly different (*p* < 0.05).

**Table 3:** Carcass composition of European sea bass fed dietary cinnamon.

Item	Initial	Cinnamon (g/kg)			
		0	10	15	20
Dry matter (%)	33.01 ± 1.12	33.72 ± 0.13	34.22 ± 0.21	33.63 ± 0.16	33.52 ± 0.13
Crude protein (%)	52.77 ± 0.23	53.58 ± 0.32	54.08 ± 0.18	53.78 ± 0.27	53.42 ± 0.15
Lipids (%)	25.77 ± 0.64	27.78 ± 0.19 *	27.55 ± 0.16 *	27.97 ± 0.33 *	28.08 ± 0.13 *
Ash (%)	16.76 ± 0.13	16.73 ± 0.25	16.83 ± 0.26	16.84 ± 0.34	17.18 ± 0.23

* Values in the same row with an asterisk are significantly different.

**Table 4:** Hematological indices of European sea bass fed dietary cinnamon.

Item	Cinnamon (g/kg)			
	0	10	15	20
Hemoglobin (g/dL)	8.99 ± 0.26 b	11.24 ± 0.36 a	9.91 ± 0.71 ab	10.86 ± 0.31 ab
Hematocrit (%)	41.23 ± 0.44 c	43.98 ± 0.41 a	45.85 ± 1.14 b	46.81 ± 0.93 ab
Red blood cells (×10^6^/mm^3^)	3.31 ± 0.04 b	4.30 ± 0.08 a	3.87 ± 0.26 a	4.13 ± 0.11 a
MCV (µm^3^/cell)	124.56 ± 1.67	102.28 ± 0.31	118.48 ± 0.47	113.52 ± 0.34
MCH (pg/cell)	27.16 ± 0.19	26.14 ± 0.68	25.61 ± 0.40	26.29 ± 0.55
MCHC (mg/dL)	21.81 ± 0.11	25.56 ± 0.01	21.61 ± 0.25	23.21 ± 0.35
White blood cells (×10^3^/mm^3^)	26.319 ± 134.14 c	28.243 ± 146.07 a	27.46 ± 118.31 b	27.60 ± 6.61 b

Values in the same row with different letters are significantly different (*p* < 0.05). MCV: mean corpuscular volume, MCH: mean corpuscular hemoglobin, MCHC: mean corpuscular hemoglobin concentration.

**Table 5:** Blood biochemical indices of European sea bass fed dietary cinnamon.

Item	Cinnamon (g/kg)			
	0	10	15	20
ALT (U/I)	82.22 ± 0.10	82.57 ± 0.07	83.24 ± 0.16	81.49 ± 0.17
AST (U/I)	80.00 ± 0.58	80.33 ± 0.67	80.67 ± 0.88	79.33 ± 0.67
ALP (U/I)	68.99 ± 0.84	74.53 ± 0.71	71.79 ± 0.67	69.30 ± 0.49
Total lipids (g/dL)	933.67 ± 4.37 c	952.67 ± 3.76 b	971.67 ± 2.03 a	960.00 ± 2.65 b
Urea (mg/dL)	4.54 ± 0.04	4.56 ± 0.15	4.66 ± 0.08	4.33 ± 0.13
Creatinine	0.61 ± 0.05	0.62 ± 0.05	0.63 ± 0.02	0.50 ± 0.04

Values in the same row with different letters are significantly different (*p* < 0.05). ALT: alanine aminotransferase; AST: aspartate aminotransferase; ALP: alkaline phosphatase.

## Data Availability

The datasets generated during and/or analyzed during the current study are available from the corresponding author on reasonable request.
